# Chronotropic Competence Indices Extracted from Wearable Sensors for Cardiovascular Diseases Management

**DOI:** 10.3390/s17112441

**Published:** 2017-10-25

**Authors:** Jiankang Wu, Jianan Li, Andrew Seely, Yi Zhu, Sisi Huang, Xiaoqin Wang, Lei Zhao, Hongliang Wang, Herry Christophe

**Affiliations:** 1 The University of Chinese Academy of Sciences, Beijing 100049, China; hlwang123@163.com; 2 Jiangsu Province Hospital, No. 200 Guangzhou Road, Nanjing 210008, China; lijianan@carm.org.cn (J.L.); lucky.zyx@163.com (Y.Z.); hss0904@163.com (S.H.); wxqsara@126.com (X.W.); zhaolei326@hotmail.com (L.Z.); 3 Ottawa Hospital Research Institute, 1053 Carling Ave, Ottawa, ON K1Y 4E9, Canada; aseely@toh.ca (A.S.); cherry@ohri.ca (H.C.)

**Keywords:** chronotropic competence indices (CCI), chronotropic incompetence (CI), cardiopulmunary exercise test (CPET), wearable sensors

## Abstract

Chronotropic incompetence (CI) has been proven to be an important factor in the diagnosis and management of cardiovascular diseases. In this paper, we extend the existing CI parameters and propose chronotropic competence indices (CCI) to describe the exercise response of the cardiopulmonary system. A cardiac chronotropic competence Test (3CT), dedicated to CCI measurement using a wearable device, is also presented. Preliminary clinical trials are presented for the validation of 3CT measurement accuracy, and to show the potential of CCI in the prevention and rehabilitation of cardiovascular diseases.

## 1. Introduction

The exercise response of the cardiopulmonary system is one of the most important basic physiological functions [1]. Cardiopulmonary exercise testing (CPET) is a widely applied clinical tool to evaluate exercise capacity and predict outcome in patients with heart failure and other cardiac conditions [2]. It provides an assessment of the integrative exercise responses involving the pulmonary, cardiovascular, and skeletal muscle systems. The major concept is to measure respiratory oxygen uptake (Vo2), carbon dioxide production (Vco2),and ventilatory measures during maximum exercise tolerance or peak exercise capacity. However, it cannot adequately reflect the cardiopulmonary system’s response through these parameters. In addition, the complex setup and difficult protocols have limited the routine use of CPET among clinicians [3]. For instance, in daily clinical practice in China, only a minority of patients are able to reach the maximum exercise tolerance and complete CPET [4].

The exercise response of the cardiopulmonary system is one of the most important basic physiological functions. Cardiopulmonary exercise testing (CPET) is a well-supported assessment technique to delineate the physiologic system(s) underlying exercise responses. The major concept is to measure the cardiopulmonary system’s exercise limit, represented by maximum oxygen consumption. The addition of ventilatory gas exchange measurements during exercise testing provides a wide array of unique and clinically useful incremental information. However, the complex setup and protocol required and difficulties in testing certain groups of patients have limited the practical use of CPET among clinicians in US [3] as well as in China, where clinicians often lack the proper training to interpret the results [4]. Exercise testing remains a remarkably durable and versatile tool that provides valuable diagnostic and prognostic information regarding patients with cardiovascular and pulmonary diseases. Clinical practice calls for an alternative to CPET which is easy to use and able to produce quantitative and meaningful measures.

In addition to the exercise testing, chronotropic incompetence (CI, defined as the inability of the heart to increase its rate commensurate with increased activity or demand) is another widely applied index in patients with cardiovascular disease. It is an independent predictor of major adverse cardiovascular events and overall mortality [5]. CI parameters or CI indices such as maximum heart rate and heart rate recovery after exercise are used to characterize CI status. They have been explored in clinical applications such as diagnosis value of coronary artery [6,7], prognosis and management of heart failure [8,9,10], diabetes [11,12,13], and hypertension [14,15]. The study of CI started in the 1970s, when Ellestad analyzed the follow-up results of 2700 patients [16], and he found that those with a slow heart rate response to exercise were at greater risk for cardiac disease than those with ischemic ST depression. Lauer et al. conducted an investigation on 1575 male participants in the Framingham Offspring Study [17]. During 7.7 years of follow-up, they found that an attenuated heart rate response to exercise, as the manifestation of chronotropic incompetence, is predictive of increased mortality and coronary heart disease incidence. Similar findings have been reported by other investigators [18,19].

In order to physiologically validate the effectiveness of CI parameters in characterizing heart exercise response, two large-scale studies were conducted. Azarbal et al. [20] reported a study on 10,021 patients who underwent single-photon emission computed tomography (SPECT) evaluated by a summed stress score together with percentage heart rate reserve, and followed up for 719 ± 252 days. They found the incremental prognostic value of percentage of heart rate reserve achieved over SPECT in the prediction of cardiac death and all-cause mortality. Later, they conducted a more comprehensive study on chronotropic incompetence, measured by the percentage of heart rate (HR) reserve achieved (HR reserve), abnormal HR recovery, reduced exercise capacity (EC), and SPECT on 11,218 patients [21]. They concluded that EC, HR reserve, and HR recovery are independent predictors of all-cause mortality and cardiac death, and add incremental prognostic value to the extent and severity of SPECT. The findings suggest that CI parameters should become the standard for assessing the adequacy of HR response during exercise testing, and that it should be routinely incorporated in risk stratification algorithms.

However, although CI parameters such as maximum heart rate at peak exercise and heart rate reserve have shown some potential, there is a lack of consistent methodology for determining CI, since the current parameters depend on the exercise types and the status of the person. To overcome this problem, some ad-hoc methods have been proposed. For instance, Wilkoff et al. [22] proposed a metric called the metabolic chronotropic relationship (MCR; also known as the chronotropic index). It is calculated from the ratio of the HR reserve to the metabolic reserve during submaximal exercise. The curves of heart rate and oxygen consumption vs. exercise intensity in CPET output are parallel for healthy individuals; MCR is usually with a 95% confidence interval between 0.8 and 1.3. However, the physiological meaning of MCR and its clinical interpretation are unclear. In addition, in order to obtain MCR or chronotropic index, a patient still has to reach peak exercise, which is often not possible in practice.

To make the CI parameter a widely usable clinical index, in this article we propose a unified formal definition of CI parameters, which we call chronotropic competence indices (CCI) [23], by adapting existing CI parameters (namely, resting heart rate, heart rate reserve, and heart rate recovery), and introducing the *chronotropic rate*, a newly defined metric. We also present a system for CCI measurement—cardiac chronotropic competence testing (3CT)—in Section 3, which is easy to use and applicable to most patients. The validation studies are given in Section 4 and Section 5.

## 2. Definition of Chronotropic Competence Indices

CI relies on heart rate response to exercise. Conceptually, this is consistent with oxygen consumption in measuring cardiopulmonary response to exercises. Based on the Fick equation [3],

(1) $$VO_{2\;max}=\left(HR\times SV\right)\times \left(C_{\left(a-v\right)}O_{2}\right)$$

where $VO_{2\;max}$ is the maximum oxygen consumption and $C_{\left(a-v\right)}O_{2}$ is the arteriovenous oxygen difference at peak exercise, HR is heart rate, and SV is stroke volume. During maximal aerobic exercise in healthy humans, the contribution to oxygen consumption of heart rate, stroke volume, and arteriovenous oxygen difference are 55%, 7.5%, and 37.5%, respectively. In a graded exercise test, the plot of oxygen consumption and heart rate against exercise intensity shows that heart rate and oxygen consumption increase to satisfy exercise needs. Thus, in measuring cardiopulmonary response to exercise, the CI parameter could be a surrogate for oxygen consumption. Therefore, we define the following four indices for the quantitative measurement of the overall profile of heart rate response during the whole exercise test.

- Resting Heart Rate $\left(HR_{rest}\right)$: The resting heart rate is defined as the heart rate when a person is awake, in a neutrally temperate environment, and has not been subject to any recent exertion or stimulation, such as stress or surprise. There is increasing evidence that elevated resting heart rate is associated with increased cardiovascular morbidity and mortality, both in the general population and in patients with cardiovascular disease [24]. The normal resting heart rate in adults is 60–80 beats per minute (bpm).
- Chronotropic Rate $\left(CR\right)$: Chronotropic rate represents the rate at which the heart rate increase as exercise intensity increases. It is measured as amount of heart rate increase in response to every unit of metabolic equivalent (MET) exercise intensity increase. In practice, it can be measured and calculated as:
  (2) $$CR=\left(HR_{stage}-HR_{rest}\right)/\left(MET_{stage}-1\right)$$
  CR is similar to the “Exercise HR” in the EACPR/AHA Joint Scientific Statement [14], where it provides insight into chronotropic competence and cardiac response to exercise. It normally increases ∽10 beats per MET.
  CR is an important parameter to provide personalized quantitative relation between HR and exercise intensity so that target heart rate (THR) can be used to prescribe exercise intensity in exercise training. However, a person’s CR may vary due to medication or rehab progress, and it is recommended to measure CR promptly or monitor CR changes in order to keep exercise prescription updated [25].
- Chronotropic Limit $\left(CL\right)$: Chronotropic limit represents the maximal heart rate an individual can achieve without severe problems through exercise stress. It is measured as Heart Rate Reserve (HRR) and calculated as
  (3) $$CL=HRR=\left(HR_{max}-HR_{rest}\right)/\left(HR_{PredM}-HR_{rest}\right)$$
  where $HR_{max}$ is the maximal heart rate one achieves during exercise test, and $HR_{PredM}$ is the predicted maximal heart rate, and is usually calculated as 220−Age.
  Maximal heart rate is usually obtained when reaching peak exercise, which is easily identified during CPET testing. In this case, the normal value of CL is 0.8–1.3. However, when CPET testing or peak exercise is not achievable, then CL normal values can be estimated from various types of exercise. For example, in a 6-min walking test, CL =0.4 for a 60 year old person should be considered normal. With a resting heart rate of 75 bpm, CR would be 10 beats per MET, and the maximal heart rate would be 109 bpm with an exercise intensity of 4.4 MET.
- Heart Rate Recovery at 1 min after Exercise $\left(HR_{recovery1}\right)$: This measure is defined as the reduction in heart rate at maximum during exercise and the rate as measured at 1 minute after stopping exercise. The measurement of $HR_{recovery1}$ does not require that the exercise intensity reaches one’s maximum capacity. The EACPR/AHA Joint Scientific Statement [14] considers that $HR_{recovery1}$ provides insight into the speed of parasympathetic reactivation, and that the normal value of $HR_{recovery1}$ should be >12 beats.
  There have been a number of clinical studies on the prognosis value of $HR_{recovery1}$. For example, Dhoble et al. [19] examined conventional cardiovascular risk factors and exercise test parameters in 6546 individuals (mean age 49 years, 58% men) between 1993 and 2003. A total of 285 patients died during the follow-up period. $HR_{recovery1}<12$ beats was found independently associated with mortality (p<0.001).

## 3. Cardiac Chronotropic Competence Test

**Figure 1:**
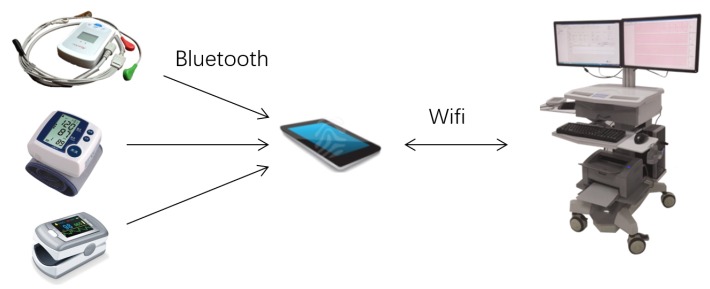
The cardiac chronotropic competence test (3CT) consists of a wearable device (**top left**), a smartphone app, and a workstation. Blood pressure meter and SpO2 meter (**bottom left**) are used to measure blood pressure and SpO2 before and after the test.

A device produced by SmartHealth Electronics Ltd. called cardiac chronotropic competence testing (3CT) was designed to measure CCI, as shown in Figure 1. 3CT consists of a wearable device, a smart phone app, and a work station. The wearable device is an ElectroCardioGram (ECG) patch with embedded 3D accelerometer and 3D gyroscope to collect activity signals. Dimensions of this device are 10.5 cm × 6.4 cm × 2.1 cm, with a total weight of 72 g.

A connector cable provides five electrodes resulting in three full-disclosure bipolar ECG channels. The digital sampling rate of the ECG signal is 250 frames per second, with an analog-to-digital (A/D) conversion of 12 bits, while the sampling rate for the 3D accelerometer and 3D gyroscope is 50 Hz. The collected ECG signals and activity signals are sent to the smart phone app via bluetooth. The smart phone app receives and processes ECG and activity signals, detects possible ECG abnormalities, derives heart rate, estimates walking step length, number of steps and walking speed, and calculates exercise intensity in terms of METs according to American College of Sports Medicine (ACSM) formula.

With 3CT, the exercise test can be done by adapting a 6-min walking protocol, or on the treadmill or on leg cycle ergometry using a standard graded exercise protocol. The 6-min walking test protocol is adapted to a 1-6-1 testing protocol: the patient is required to stay at resting status for 1 min in order to properly obtain resting heart rate, blood pressure, and other measures. The smart phone app then gives instruction to the patient to start walking, and subsequently every minute a voice instruction to walk as quickly as possible if there is no ECG abnormality detected. The patient can know their walking speed and the distance they have covered from the app interface. After completing 6 min of walking, the patient is required to stop walking and stay at rest for at least 1 min. 3CT then measures the heart rate recovery $HR_{recovery1}$.

**Figure 2:**
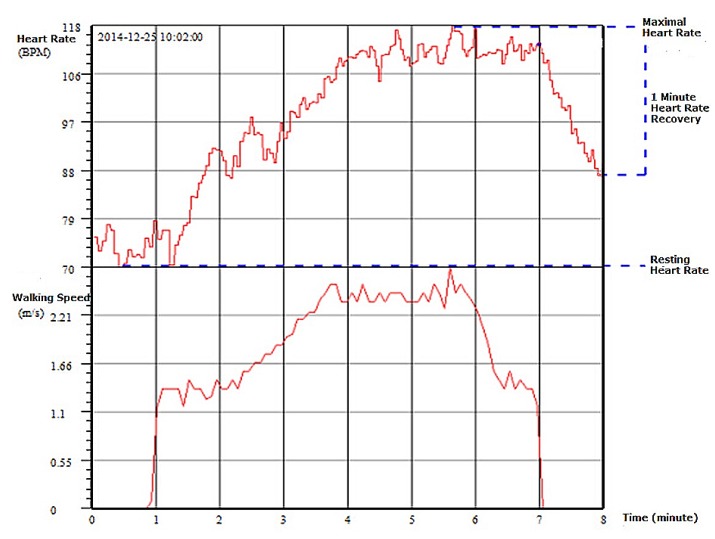
Curves of heart rate variation against exercise intensity during the entire 8 min walking test.

Throughout the whole testing process, the smart phone app processes signals in real-time, gives testing instructions, and sends all data and testing information to the work station. At the work station, the clinician can observe all signals and status, and sends instructions to the smart phone app. At the end of testing, 3CT sends a report to the clinician for approval. The report consists of a form of CCI values as shown in Table 1, together with curves of heart rate variation against exercise intensity during the whole 8 min (as shown in Figure 2), and abnormal ECG signals (if any).

## 4. Validation of 3CT

Since the measurement accuracy of CCI by 3CT depends on its heart rate and walking speed measurement. A 3CT heart rate measurement validation trial has been conducted to compare 3CT equipment with the CPET machine manufactured by Care Fusion, Master Screen-CPET with software version 5.0. Subjects are required to wear ECG leads from both CPET and 3CT, and to walk on the CPET treadmill using the 3CT 1-6-1 minutes protocol. The walking speed during 6 min was 5–6 km per hour. In this setting, both 3CT and CPET ECG devices were under the same condition. The heart rate measurement accuracy of the ECG device depends on the noise level due to exercise.

Walking distance measurement validation was done by the standard 6-min walking setting: the subject walked between two poles 100 feet apart for 6 min, and the walking distance displayed by 3CT was compared with manual marks on the ground.

### Validation Results and Analysis

In accordance with the ethics policy of Jiangsu Provincial Hospital, 32 healthy volunteers aged between 50 and 65 were recruited, signed an informed consent, and participated in the validation test.

**Figure 3:**
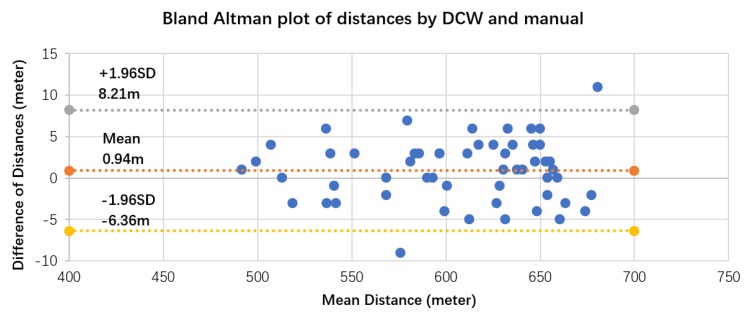
Bland–Altman plot for distance data measured by 3CT and manually measured, with the representation of bias (0.94 m) and limits of agreement (dotted line) from −1.96SD (−6.36 m) to 1.96SD (8.21 m).

3CT generates walking speed by estimating each step length based on 3D accelerometer and 3D gyroscope data, and accumulates all distances covered by steps. In the walking distance measurement validation test, each subject completed two 6 min walks; there were a total of 64 tests. The distance measurement accuracy was verified at the last cycle, where the subject stopped in between two cones placed at the beginning and end of the 30 m. A Bland–Altman plot was used to compare 3CT distances against manually measured distances. As in Figure 3, the *x*-axis is the mean of two distances, and the *y*-axis is the difference of the two distances. The systematic bias of 3CT against manual measurement was 0.94 meters; 95% of the difference in distances fell between −6.36 m and 8.21 m.

**Figure 4:**
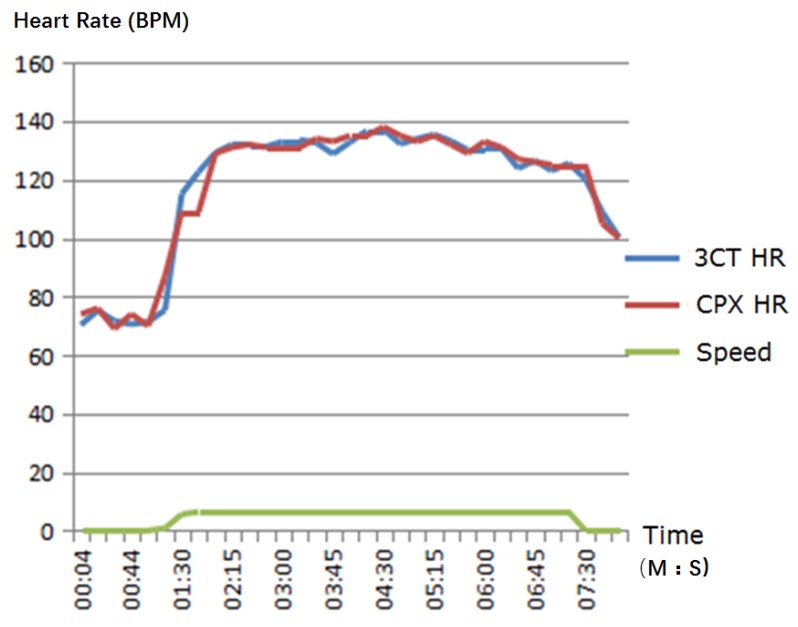
Heart rate comparison along 8 min of exercise test time.

In the heart rate validation clinical trial, some subjects did not have experience using a treadmill, and this resulted in large ECG noise. The average heart rate has been considered by averaging over a time period. The reference CPET produces a heart rate by averaging over 15 s. If the acquired ECG signal is too noisy to produce a reasonable heart rate, the system assumes no heart rate change and the heart rate of the last 15 s interval is used for the current. On the contrary, 3CT produces a heart rate every 5 s. For the purpose of consistency, three 3CT heart rates were averaged to generate one heart rate. However, there is still an issue of time synchronization between reference CPET and 3CT when comparing heart rate. Figure 4 shows the heart rate comparison for a subject. The two systems produce consistent heart rate during exercise test, although there are time synchronization differences. For CPET, from time 01:30 to 01:45, the heart rate curve is flat. This is due to large noise—the system can only assume no heart rate changes.

The heart rate mean error was calculated by averaging for the whole testing time for each subject and then for all 32 subjects. The mean error and deviation were 0.40 ± 1.43 bpm. The mean error and deviation of chronotropic rate (CR) produced by CPET and 3CT were 0.36 ± 1.32 bpm/MET.

**Figure 5:**
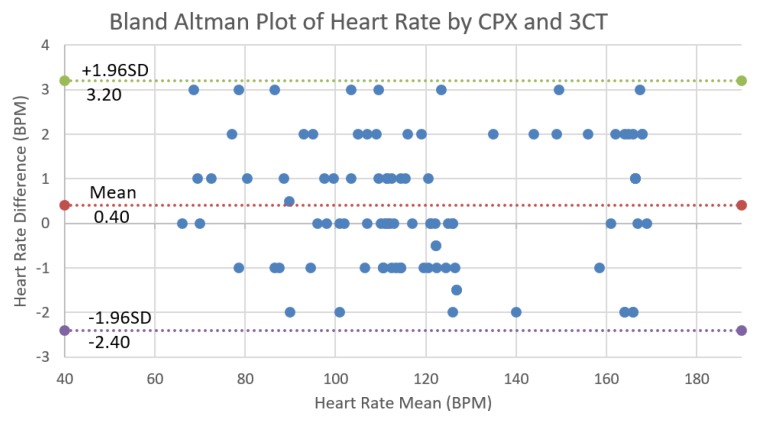
Bland–Altman plot for 99 heart rate data pairs of three subjects measured by cardiopulmonary exercise testing (CPET) and 3CT, with the representation of bias 0.40 and limits of agreement (dotted line) from −1.96SD (−2.40) to 1.96SD (3.20). Here, three subjects are empirically picked up and plotted in the figure for easy comparison.

Figure 5 is the Bland–Altman plot for the heart rate data pairs measured by CPET and 3CT during the exercise test. The systematic bias was 0.40 bpm with a 95% confidence interval between −2.4 and 3.2 bpm. The validation results show that 3CT had clinically acceptable measurement accuracy on chronotropic competence indices. Its advantages of ease to use, simple maintenance and operation, and clinical usefulness of CCI make 3CT ideal equipment in cardiac exercise test in hospitals, clinics, and even community, contributing to the rehabilitation and prevention of cardiopulmonary diseases.

## 5. CCI for Cardiac Rehabilitation Validation

A clinical trial was designed to assess the effectiveness of digital rehabilitation delivered to home-based interventions on motor and non-motor outcomes after stroke. Before the intervention, all participants signed an informed consent approved by the institutional ethics policy review board of the Jiangsu Provincial Hospital. Sixty-one participants were recruited from the Nanjing metropolitan area. The main criteria for eligibility included a unilateral ischemic or hemorrhagic stroke within the previous 6 months, with some voluntary movement and preserved cognitive function. The main exclusion criteria were lack of independence before the stroke and antispasticity injection in the hemiparetic upper-extremity since stroke onset. Additional exclusion criteria were adopted to ensure that potential participants could successfully complete study-related interventions without the direct supervision of a therapist.

After meeting inclusion criteria, participants completed baseline evaluation. All evaluations were completed by a physical therapist or occupational therapist. The baseline evaluation items included Mini-Mental State Examination (MMSE) [26], Health Survey questionnaire SF-36 [27], ADL Barthel Index [28], Rankin scale [29], Motricity Index [30], Endurance, walking ability, walking stability, and self-rated depression scale. After baseline testing, patients were randomly assigned to rehab group and control group with a random number-generated assignment.

Each patient from both groups were evaluated at the beginning and after 3 months using both subjective/qualitative and objective/quantitative measures—namely, the International Classification of Functioning, Disability and Health (ICF), and chronotropic competence indices and 6 Minute Walking Test (6MWT) distance.

Patients in the control group were given personalized rehab advice after the baseline test. There were no home rehab services for control group patients. Patients in the rehab group were equipped with a MicroSens rehab assistant, which consists of a rehab app on a smart phone and a wearable device. Based on the evaluation results, the physical therapist or occupational therapist designed a rehab prescription for each patient [22,24], and explained to the patient when and how to use the MicroSens rehab assistant to perform rehab exercises. The rehab exercise prescription was entered into the rehab app in terms of exercise time, duration, and target heart rate. The chest-worn device captures one lead ECG and movement signal and sends data to the rehab app via Bluetooth. The app then processes ECG and movement signals, detects heart rate and abnormal ECG events, classifies posture, estimates the exercise intensity when walking or running, and detects falls. Based on the rehab exercise prescription, the app reminds the patient to start rehab exercise on time and to maintain exercise intensity by comparing current heart rate to target heart rate. The app also warns of any detected abnormal heart rhythms. In addition, the app sends waveform signals, processes results, and sends rehab status to a server, where physical therapists or occupational therapists and family members can monitor the rehab process, deal with possible events, and generate rehab reports.

There were 30 patients in the rehab group who completed the whole rehab process, and 31 patients in the control group. Here we mainly care about exercise capability improvement of stroke patients after 3 months of regular exercise training at home. For the purpose of the evaluation of exercise capabilities, we chose four activity-related parameters from ICF [31]; namely, d450 walking, d640 doing housework, d710 basic interpersonal interactions, and b730 muscle power, as the subjective/qualitative measures. Three objective/quantitative parameters from 3CT were chosen; namely, 6 MWT distance, chronotropic rate, and heart rate recovery after 1 min. The purpose of the trial was also to verify the consistency between these two types of evaluation systems.

Table 2 and Table 3 compare results between the control and rehab group after 3 months, under the ICF subjective evaluation system and the 3CT objective evaluation system. Significance was determined at the 5% level via *t*-test. In Table 2, after 3 months the control group did not show significant improvement with respect to walking (p=0.0563), doing housework (p=0.1607), or interpersonal interactions (p=1.0000), while the rehab group showed significant improvement after 3 months rehab training with respect to these three ICF measures (p=0.0000, 0.0005, and 0.0007, respectively). With respect to ICF B730 muscle power, both control group and rehab group showed significant improvement with p=0.0314 and p=0.0117. Comparison between control and rehab groups after 3 months using *t*-test shows that the rehab training group gained significantly in all four ICF measures (p=0.0070, 0.0209, 0.0089, and 0.0000, respectively).

Table 3 compares the results at 3 months between control and rehab groups under the 3CT objective evaluation system. After 3 months, the control group did not show significant improvement on the 6-min walking distance, chronotropic rate, or 1 min heart rate recovery (p=0.152, 0.753, and 0.557, respectively), while the rehab group did (p=0.0006, 0.025, and 0.002, respectively). Three-month rehab training made the rehab group significantly better over the control group with respect to all three 3CT objective measures (p=0.0445, 0.0121, and 0.0414, respectively).

We also observe from Table 2 and Table 3 that both ICF subjective evaluation measures and 3CT objective evaluation measures are consistent in this clinical trial data analysis.

## 6. Conclusions and Remarks

Chronotropic incompetence (CI) has been widely studied since 1970, and has been proven to be an important cardiovascular risk factor which is closely related to major cardiovascular diseases, and has great potential in clinical applications. Here we have proposed chronotropic competence indices (CCI) with the purpose of unifying the definition and clinical interpretation and provide the overall profile of heart rate response to exercise process. Cardiac chronotropic competence testing (3CT) is presented, which is dedicated to the measurement of CCI. By adapting a 6-min walking test protocol, the 6 MWT distance and CCI constitute a quantitative evaluation system for the cardiovascular system, especially exercise capabilities.

We have also reported a clinical trial for the validation of 3CT, and a clinical trial on the use of CCI in cardiac rehabilitation applications. Both clinical trials are preliminary, and have shown the potentials of CCI and 3CT in cardiac exercise test and cardiac rehabilitations and prevention. The sound scientific basis and clear interpretations of CCI make it widely acceptable. 3CT is very easy to use, simple to maintain, and therefore has the potential to play an important role in the popularization of the cardiac exercise test. This will have a great impact in cardiovascular disease rehabilitation and prevention.

Further studies are required to explore the physiological and pathological meanings of CCI. We are planning more clinical studies to investigate CCI applications in cardiac rehabilitation for coronary heart disease patients and heart failure patients.

## Figures and Tables

**Table 1:** 3CT chronotropic competence indices (CCI) report form.

Chronotropic Competence Indices	Measured	Reference Value
Resting heart rate (HR)		60–80 bpm
Max HR in Exercise		220−age
Metabolic equivalent (MET) at Max HR		
Walking distance and grading		I ≤ 300 m < II ≤ 375 m < III ≤ 450 m < IV
Chronotropic rate		∽10 BPM
Chronotropic limit		
HR recovery after exercise		≥12 BPM

**Table 2:** Comparison of 3-month rehab results between control group and rehab group under International Classification of Functioning, Disability and Health (ICF) subjective evaluation system.

		d450 Walking	d640 Doing Housework	d710 Basic Interpersonal Interactions	B730 Muscle Power
Control group	mean before trial	0.7097	0.3548	1.2258	1.2903
	variance before trial	0.8638	0.6082	1.0555	0.5287
	mean after 3 months	0.5161	0.4194	1.2258	1.0968
	variance after 3 months	0.7690	0.6204	0.9903	0.5975
	significance of improvement	0.0563	0.1607	1.0000	0.0314
Rehab group	mean before trial	2.6667	3.8000	1.0667	2.8000
	variance before trial	0.7112	1.1552	0.7303	0.8550
	mean after 3 months	1.4667	3.0000	0.4000	2.4000
	variance after 3 months	0.3317	0.3371	0.2598	0.4052
	significance of improvement	0.0000	0.0005	0.0007	0.0117
Significance of improvement of rehab group vs. control group		0.0070	0.0209	0.0089	0.0000

**Table 3:** Comparison of 3-month rehab results between control group and rehab group under 3CT objective evaluation system. CR: chronotropic rate.

		6 MWT (meter)	CR (BPM/MET)	$HR_{recovery1}$(BPM)
Control group	mean before trial	225.29	12.56	24.75
	variance before trial	118.54	5.11	11.47
	mean after 3 months	234.14	12.37	26.64
	variance after 3 months	129.52	4.46	12.85
	significance of improvement	0.152	0.753	0.557
Rehab group	mean before trial	210.12	11.03	21.21
	variance before trial	139.63	3.03	11.09
	mean after 3 months	234.17	12.41	28.10
	variance after 3 months	153.81	3.82	13.40
	significance of improvement	0.0006	0.025	0.002
Significance of improvement of rehab group vs. control group		0.0445	0.0121	0.0414
